# Flype: Integrating Molecular and Pharmacogenomic Results to Enhance Oncology Patient Care in a Community-Based Academic Cancer Center

**DOI:** 10.3390/cancers18162560

**Published:** 2026-08-10

**Authors:** Donald L. Helseth, Nicholas Miller, Mathew Yang, Henry Wittich, Qin Zhao, Tom Werth, Linda M. Sabatini, Mir Alikhan, Megan Parilla, Amandeep Kaur, Xiaoyan Yang, Kathy A. Mangold, Michael Bouma, Henry M. Dunnenberger, Dyson T. Wake, Annette Sereika, Gayathri Moorthy, Peter J. Hulick, Karen L. Kaul, Janaradan D. Khandekar

**Affiliations:** 1 Neaman Center for Personalized Medicine, Endeavor Health, Evanston, IL 60201, USAmathew.yang@endeavorhealth.org (M.Y.); henry.wittich@endeavorhealth.org (H.W.); qin.zhao@endeavorhealth.org (Q.Z.); mark.dunnenberger@endeavorhealth.org (H.M.D.); dyson.wake@endeavorhealth.org (D.T.W.); annette.sereika@endeavorhealth.org (A.S.); gayathri.moorthy@endeavorhealth.org (G.M.); peter.hulick@endeavorhealth.org (P.J.H.); janardan.khandekar@endeavorhealth.org (J.D.K.); 2 Health Information Technology, Endeavor Health, Skokie, IL 60077, USA; thomas.werth@endeavorhealth.org; 3 Department of Pathology, Endeavor Health, Evanston, IL 60201, USA; linda.sabatini@endeavorhealth.org (L.M.S.); mir.alikhan@endeavorhealth.org (M.A.); megan.parilla@endeavorhealth.org (M.P.); amandeep.kaur@endeavorhealth.org (A.K.); xiaoyan.yang@endeavorhealth.org (X.Y.); kathy.mangold@endeavorhealth.org (K.A.M.); michael.bouma@endeavorhealth.org (M.B.); karen.kaul@endeavorhealth.org (K.L.K.)

**Keywords:** bioinformatics, pharmacogenomics, precision oncology, molecular tumor board, next-generation sequencing, NGS

## Abstract

**Background/Objectives:** We describe how our in-house bioinformatics platform, Flype, has evolved from being a variant repository to an enterprise role as an electronic medical record (EMR) content provider, powering molecular pathology reporting, pharmacogenomics reporting, sending discrete data to our EMR, aggregating internal and external molecular test results and powering our molecular tumor board (MTB). **Methods:** In response to critical pain points, we developed Docket, a sample tracking system in Flype, which manages multiple individual in-house molecular tests for NGS assays, pharmacogenomic (PGX) assays and additional molecular testing. To help with interpretation and integration of all internal and external assays, we developed a clinical outcomes view in Flype. To improve the efficiency of our molecular pathologists reporting results, we developed Convo 2.0, which integrates OncoKB interpretations and other information. Flype can also be used by our pathologists to submit patient molecular results to NCI’s ComboMATCH and retrieve clinical trial recommendations. **Results:** Flype was used during our Kellogg Cancer Genomic Initiative for reporting PGX integration, MTB review and integration of EMR prescription information with internal and external molecular test results. Integrating PGX results led to several recommendations against the use of drugs metabolized by, for example, *CYP2D6* or *TPMT*, along with warnings about altered pain relief. Enhancements in report sign-out and the use of file transfer scripts have led to reduced turnaround time. **Conclusions:** Flype supports hundreds of users performing different roles in molecular diagnostics. We discuss lessons learned adapting our software to support continuously changing test requirements.

## Simple Summary

Integration of pharmacogenomic results with an oncology patient’s tumor molecular test results using Flype, a software developed at our institution, led to the identification of actionable genomic alterations to determine therapy options in patients with advanced cancers for whom standard therapies had been exhausted. Software enhancements to Flype supported the Kellogg Cancer Genomic Initiative, improved molecular test report turnaround times, while providing tools for molecular tumor board patient reviews. Integrating pharmacogenomic results into the molecular tumor board review led to improved therapeutic efficacy, such as avoiding the use of tamoxifen due to an increased risk of treatment failure and better pain management by detecting reduced *CYP2D6* activation.

## 1. Introduction

We wish to provide an update on the continuing development of Flype, our repository for next-generation sequencing (NGS) test results, report generation tool, knowledge base, pharmacogenomics (PGX) software and other molecular diagnostic tools which power personalized medicine at our institution [1]. Flype is an integral part of our institution’s genomic learning healthcare system (gLHS) [2] and is often referred to as our “Swiss Army knife”, capable of adapting to new requirements for personalized medicine. Since inception, Flype has evolved from its original role as a variant repository to an enterprise role as an electronic medical record (EMR) content provider, powering molecular pathology reporting, pharmacogenomics reporting, sending discrete data to our EMR which gets made into genomic indicators to trigger interruptive alerts to reduce adverse drug events, aggregating internal and external molecular test results and powering our molecular tumor board (MTB). This communication is intended to help illustrate how we have adapted and extended our software in response to the continuously evolving needs of our institution in the hopes our examples will offer insight to other institutions struggling with implementing similar projects. Specifically, in response to critical pain points identified by our molecular pathology lab, we developed Docket, an integrated laboratory information management system (LIMS) sample tracking system in Flype. Docket now helps manage multiple molecular tests for NGS assays, PGX assays and additional molecular testing. To support our institution’s molecular tumor board (MTB) reviews, we developed an MTB note generation tool with a report view used to host and present cases during MTBs. Additionally, to help evaluate our patient’s molecular needs, we have implemented a clinical outcomes view of their molecular test results which aggregates and summarizes both internal and external molecular test results and displays them along with diagnostic and treatment information pulled from our enterprise data warehouse for each patient. Integrating PGX results with tumor molecular test results for review during MTBs should improve the accuracy of treatment, avoid adverse effects and provide better pain management to patients. To improve the efficiency of our molecular pathologists, we added an enhanced knowledge base, Convo 2.0, and integrated OncoKB [3,4] through their application programming interface (API), licensed through Memorial Sloan Kettering (MSK), to pre-populate our molecular report generation tool and enforce structured disease nomenclature. Flype also allows our molecular pathologists to submit patient molecular results to NCI’s ComboMATCH [5], retrieving clinical trial recommendations for our patients. We have extended Flype for use by our genetic counselors and added links to Flype for pathologists to refer patients for genetic counseling. We conclude with a discussion of lessons learned adapting Flype to support continuously changing test requirements.

## 2. Materials and Methods

The software, Flype [1], is a web-based platform that has been built over the past ten years by a small team of bioinformaticians at our community-based academic cancer center and, as such, has undergone extensive customizations which are unique to our environment. As recently described [6], the enhancements and customizations to the original Flype architecture described in this document, including the development of Docket as a laboratory information management system, limits the ability of individual modules of Flype to be used in other settings. Flype is built using Django [7], a popular web application framework written in Python 3.12, which allows us to create separate project folders for different modules. Since our original publication, we have upgraded Django to a more recent long-term support version (4.2). This provided security updates and allowed better integration of pre-existing Django modules. For example, our bioinformatics team has implemented a local stand-alone instance of Django-wiki [8] to share tips, best demonstrated practices and other development notes within the team and are planning to integrate it within Flype to provide links to training and documentation. Flype was used to empower our recently completed Kellogg Cancer Genomic Initiative (KCGI) at Endeavor Health [6,9], leading to the successful adoption and integration of personalized data within our health system.

No generative artificial intelligence (GenAI) was used in the preparation of this paper.

## 3. Results

### 3.1. Docket

#### 3.1.1. Sample Tracking and Result Integration

Our sample tracking module, Docket, arose because of our need to ensure multiple independent molecular tests were returned for all internally analyzed samples. We were routinely using the Ion Torrent Oncomine 161 plus Oncomine 409 assays (Thermo Fisher Scientific, Waltham, MA, USA) and running the ArcherDx (Integrated DNA Technologies, Inc., Coralville, IA, USA) translocation assay on certain tumor types. Docket helps coordinate the reporting of these three separate assays as one final report. In addition, we run the Ion Torrent Heme 84 gene panel for blood cancers and the Ion Torrent HotSpot 50 gene panel for samples without sufficient DNA for the larger Oncomine panel. Unlike tools like SIMPL [10], we designed Flype for automated sample input from PowerPath (Sunquest Information System, Tucson, AZ, USA), the sample tracking system used by our Pathology department, using Linux cron jobs to populate Docket every three hours or upon demand through the Docket Admin page.

**Figure 1:**
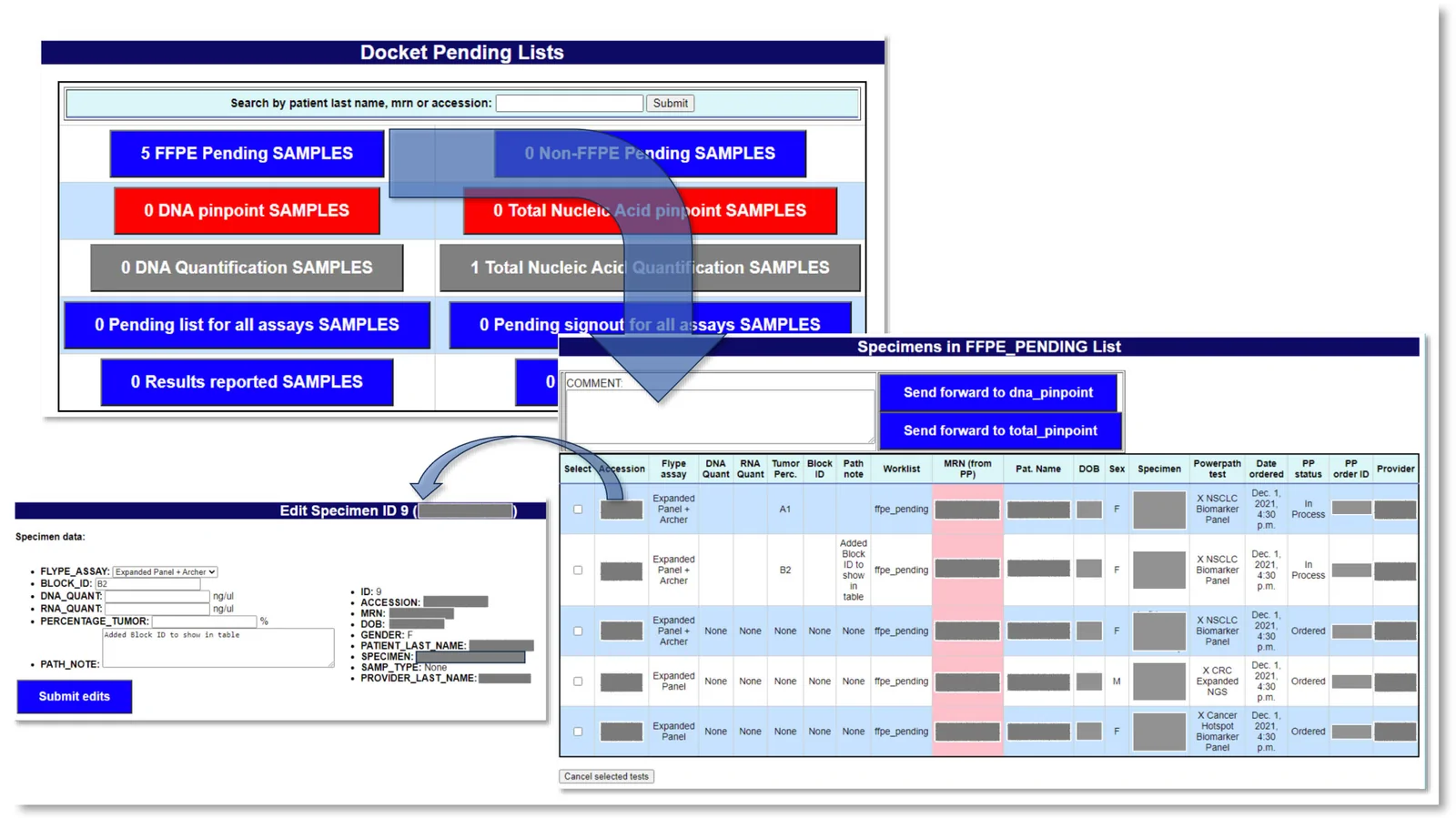
Docket pending lists which drill down to a list of pending samples in each category. Clicking an accession (obscured for patient privacy) leads to a sample detail page where pathologists can add notes and medical laboratory scientists can add DNA/total nucleic acid quantitation which is used in subsequent steps.

**Figure 2:**
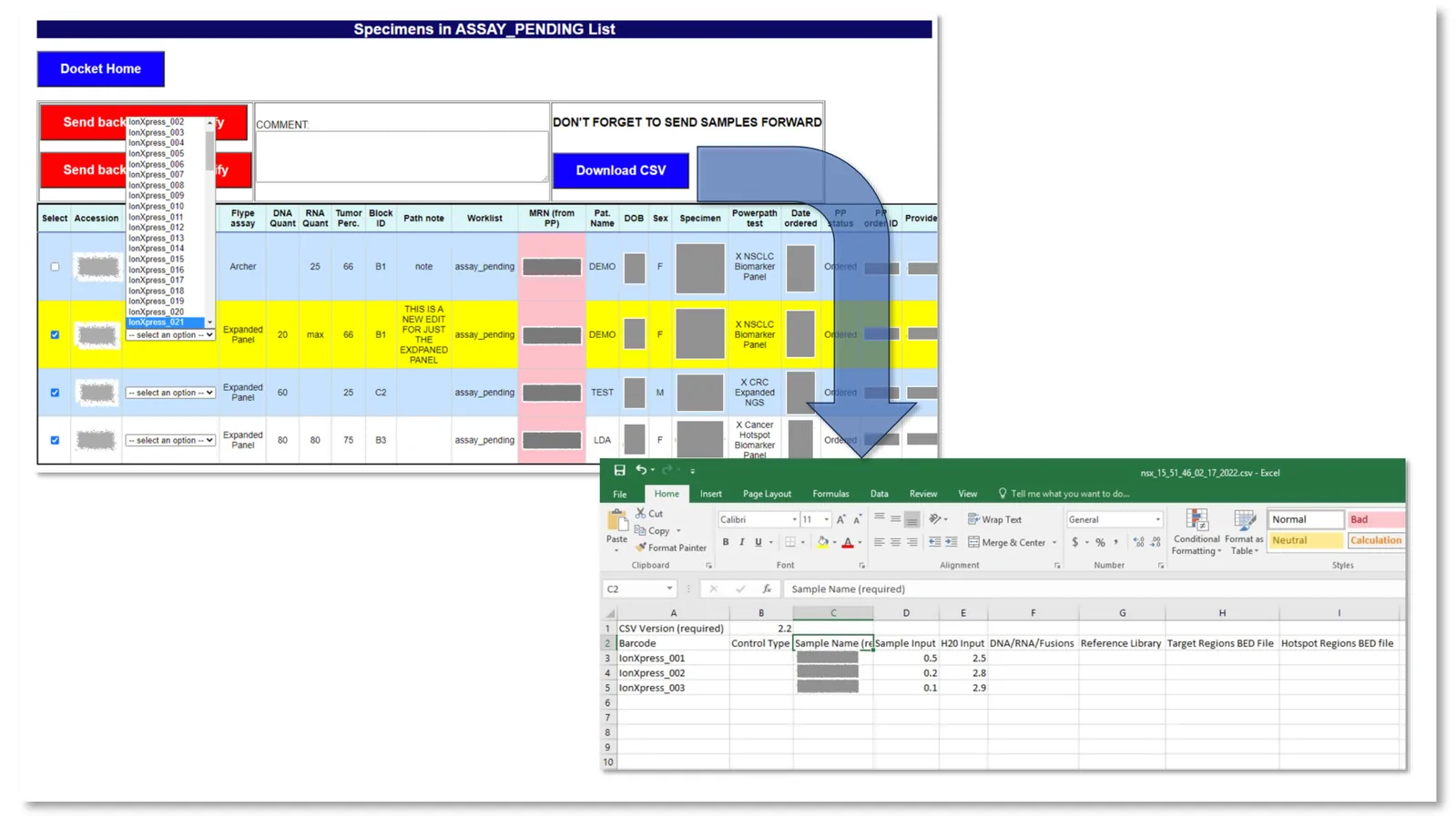
After quantitation, medical laboratory scientists select samples for inclusion on an upcoming run, assign barcodes and generate a CSV file from Docket for uploading to the sequencer.

Beyond tracking sample status, we developed Docket to help our molecular diagnostics lab record sample quantitation and generate comma-separated values (CSV) worksheet files for upload to the DNA sequencers to minimize data entry errors and eliminate the “who has today’s sample worksheet” problem in the lab. Lab members can post notes to a sample message board in Docket and pathologists can add their sample-specific notes or change the test if there is insufficient sample for the larger panel. Docket has pending lists for samples needing DNA pinpointing or total nucleic acid pinpointing, DNA or total nucleic acid quantification, pending lists for all assays, samples pending sign-out and samples reported, canceled or needing repeat. Docket can be searched by patient accession, medical record number (MRN) or patient name to find samples in all stages of processing (Figure 1). After DNA or total nucleic acid quantitation has been entered in Docket, medical laboratory scientists select samples to include on a run, assign barcodes and download a CSV file to upload to the Ion Torrent sequencer (Figure 2).

Docket is used to highlight NGS samples which have been signed out but are still missing test results such as an ArcherDx translocation assay, and this status is reflected on the analysis home page as “Signed out. Archer pending”. Pathologists reviewing a sample’s results can mark it for rerun from the Flype analysis dashboard, which updates the sample’s Docket record, sending the sample back to the appropriate worklist for repeat sequencing. Docket has a field labeled “Bridge Note” that is visible in a dialog box on the Flype results dashboard where pathologists reviewing a sample can add a note to send back to the Docket sample information (an example from an Oncomine 161 assay was *“Repeat 161 for uniformity issue—Looks like pool 2 is missing from 161 run”*) and a button “Please repeat sample” which sends the sample back to the appropriate Docket worklist and changes the display on the analysis home so other pathologists reviewing pending samples know a repeat was requested.

To improve the accuracy of importing Archer results into Flype, we developed a web page that allows lab members to upload ArcherDx results (a TSV downloaded from our local ArcherDx portal; Archer Analysis Version 6.2.7). This updates the Docket records for all accessions on a particular ArcherDx assay, updating the corresponding analysis status to alert the pathologists that the tests are completed so they can prepare an amended report incorporating the ArcherDx results. The new ArcherDx results appear at the bottom of the corresponding sample dashboards with the option for the pathologist to include them in the report or add a comment if nothing significant was found and then amend the report. Capturing the ArcherDx output and uploading it directly eliminates the need for manual entry of chromosomal rearrangements. Any ArcherDx result selected by the pathologist for reporting will appear alongside variants, copy number [11], tumor mutation burden (TMB), microsatellite instability (MSI) and other results on the final report.

We have recently begun displaying ArcherDx as a separate, stand-alone assay type in Docket. This will allow better tracking of ArcherDx turnaround times, separate from other NGS assays, while still ensuring the ArcherDx results are reported with the correct Oncomine or Hotspot sample and will allow future reporting of just ArcherDx for cases where this test is requested separately.

Pathologists or lab members can quickly view samples at any stage of analysis by assay type, and the lab manager can use Docket to help manage daily staff allocation. We provide a permissioned Docket landing page for lab members with the ability to navigate to other portions of Flype based upon their permissions. Docket provides a sample tracking dashboard to help lab managers track sample turnaround time. Docket also has a permissioned Admin page where the lab manager can enter reagent lot information, flagging expired lots, and can manually query PowerPath.

#### 3.1.2. Docket for Pharmacogenomics

Docket was extended to support our in-house PGX testing. This testing currently involves a single nucleotide polymorphism (SNP) array panel analyzed using a customized Agena Veridose panel (Agena Bioscience, San Diego, CA, USA) combined with custom assays for *CYP2D6* copy number changes and other relevant non-SNP changes in PGX genes. Docket generates six separate CSV files as a part of the sample workflow, minimizing the risk of accession transcription errors and keeping track of SNP array assays (10 per plate + 2 controls), *CYP2D6* copy number analysis that mirrors the SNP array setup and ancillary assays (15 per run). Docket updates the sample status when results are uploaded to Flype using the permissioned PGX upload page, allowing our lab to keep track of samples needing repeats and eliminating the need to manually update a “pending list” based upon an email when samples are signed out. When the pharmacogenomicist reviews a sample in Flype and decides that either the SNP array or ancillary assays need to be rerun, they select that from the PGX sign-out page, sending the sample back to Docket marked for rerun and updating the analysis home page showing the sample was marked for rerun. Docket also allows us to better track both NGS and PGX sample turnaround times and prioritize samples needed for cancer treatment as a part of our recently completed KCGI at Endeavor Health [6,9]. We have also used our Docket PGX view in Flype to check whether an internal PGX order is a duplicate, reducing lab time and expense by unnecessarily extracting DNA and running repeat testing.

**Figure 3:**
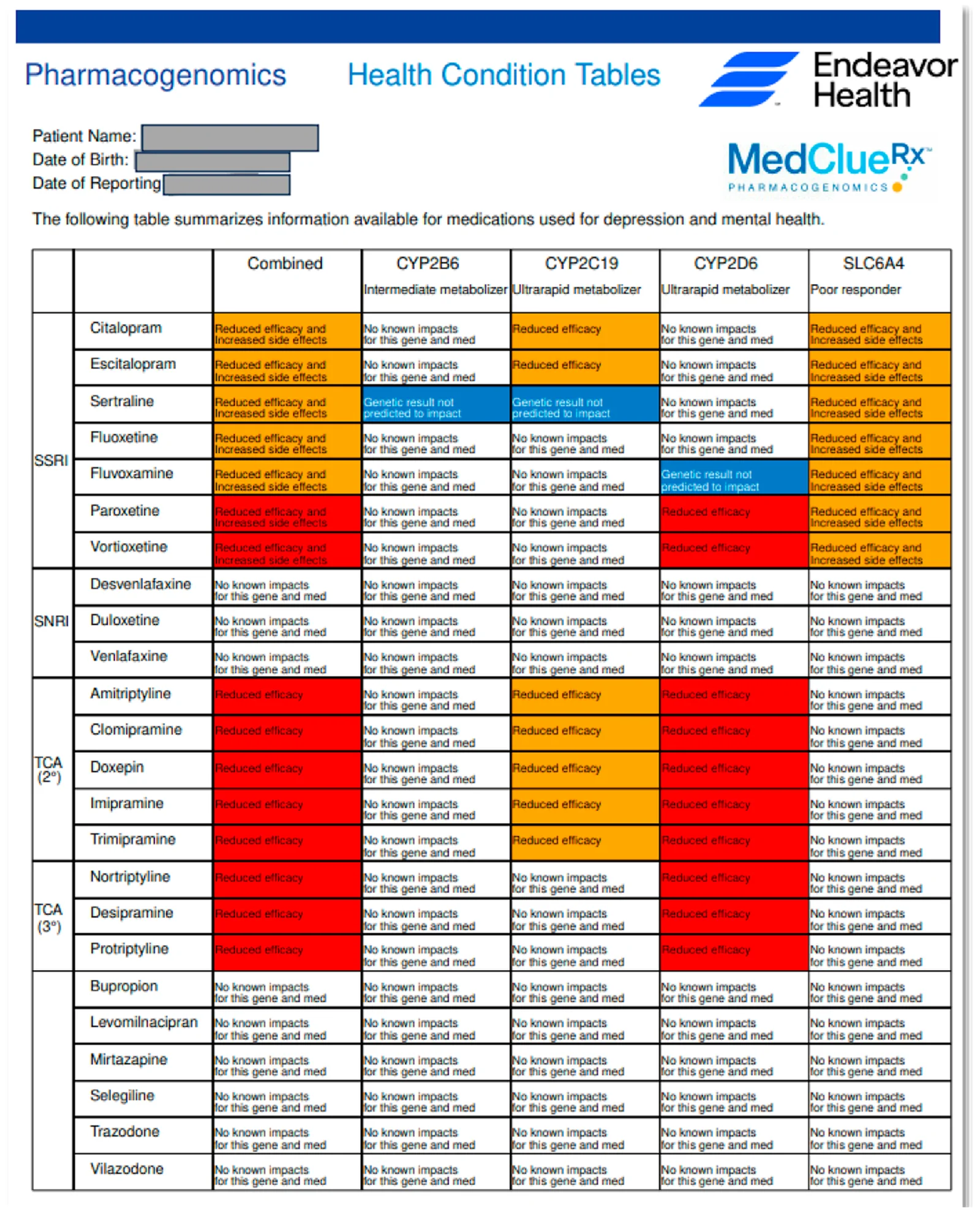
Example Health Conditions Table from a patient’s pharmacogenomics report summarizing the impact of their variants on psychiatric medications. Color coding and categories are summarized on the preceding page in the patient’s report. Similar impact summaries are being developed in conjunction with our cardiology department. Red means “Reduced Efficacy and/or Increased Side Effects” (with a significant increased risk of side effects), orange means “Reduced Efficacy and/or Increased Side Effects” (these are medications to which you may not respond as well compared to the average patient), blue means “Genetic result not predicted to impact”, and white means “No known impacts for this gene and medication”.

Flype generates PGX reports as PDFs and submits them to our EMR and, since 2016, Flype has been generating discrete data involved in interruptive alerts to reduce adverse effects [1,12]. Using Flype’s modular approach, we were able to add a parallel knowledge base with Spanish-language translations, allowing for the generation of ad hoc PGX reports in Spanish. Following the initial launch, we are currently working on system improvements including (1) automated ingestion of patient’s preferred language to automate identification of Spanish-speaking patients and (2) functionality to allow for the submission of both English and Spanish language reports in a manner accessible to both patients and providers. Similarly, working with different clinical departments within our organization, we have used Flype to include specific psychiatric PGX panel reporting and are developing a cardiovascular PGX report view. The psychiatric panel is presented as a “Health Conditions Table”, showing categories like Selective Serotonin Reuptake Inhibitors (SSRI), Serotonin-norepinephrine reuptake inhibitors (SNRIs), and two classes of Tricyclic antidepressants (TCAs). We include summaries for specific genes (currently *CYP2B6*, *CYP2C19*, *CYP2D6* and *SLC6A4*) and combined interpretation across categories related to depression and mental health. These summaries are available to patients and ordering providers (see Figure 3).

#### 3.1.3. Migration Toward EPIC Beaker

Since the last Flype paper [1], our institution has expanded from four to nine separate hospitals. Our Health Information Technology (Health IT) department is in the process of implementing a single common EPIC (Verona, WI, USA) platform across all hospitals. When that is achieved later this year, we will migrate clinical ordering from PowerPath (and SoftLab, SCC Soft Computer, Clearwater, FL, USA) to EPIC’s Beaker. Our bioinformatics team is working with our Health IT organization to ensure none of Docket’s features will be impacted by moving to Beaker; since all that is changing is where our ordering data is coming from, we built a middleware layer before Docket to process the Beaker orders. At present, we anticipate continuing to rely on the features in Docket for several years after our enterprise EPIC integration and PowerPath to Beaker migration goes live.

### 3.2. Powering Our Molecular Tumor Board

**Figure 4:**
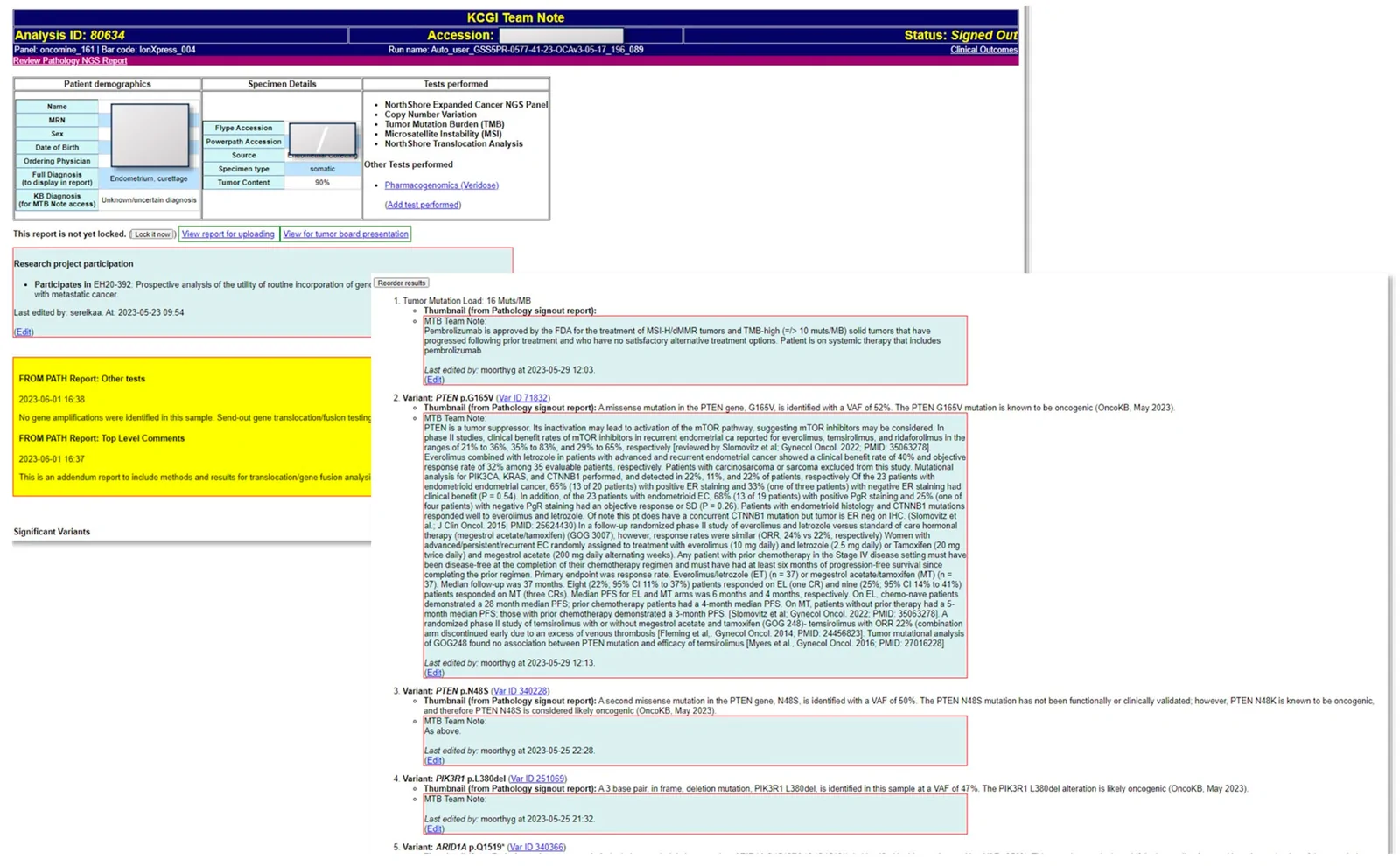
MTB note preparation—After an NGS test is signed out by our Molecular Pathology group, members of our KCGI team (pharmacists and APNs with oncology and molecular biology training) identify treatments based on actionable variants, assembling their notes and using Flype to present their recommendations at the institutional MTB.

**Figure 5:**
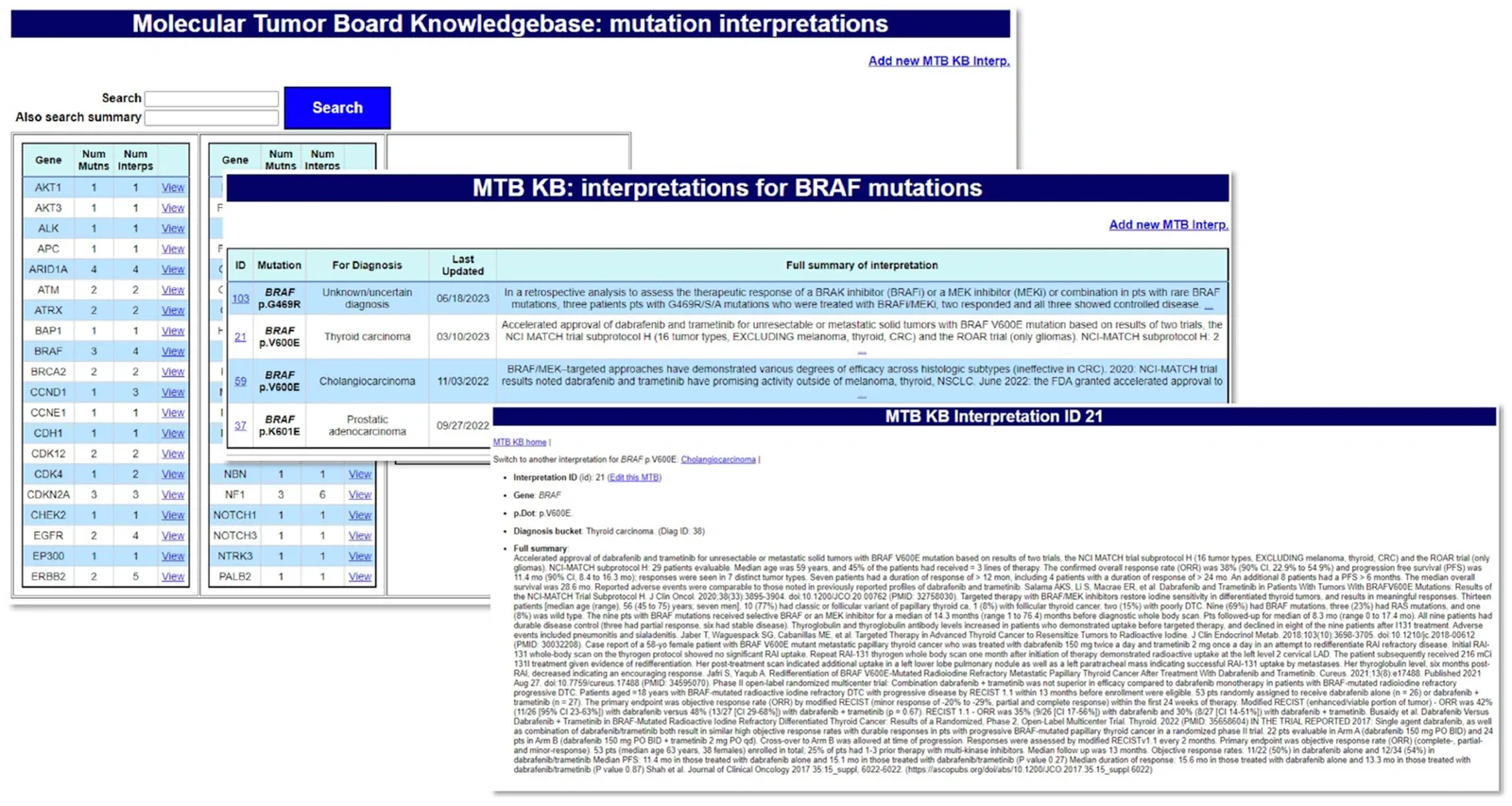
MTB Knowledge Base—The MTB knowledge base is populated during MTB note generation and allows the reuse of gene/variant/diagnosis-specific variant interpretations.

**Figure 6:**
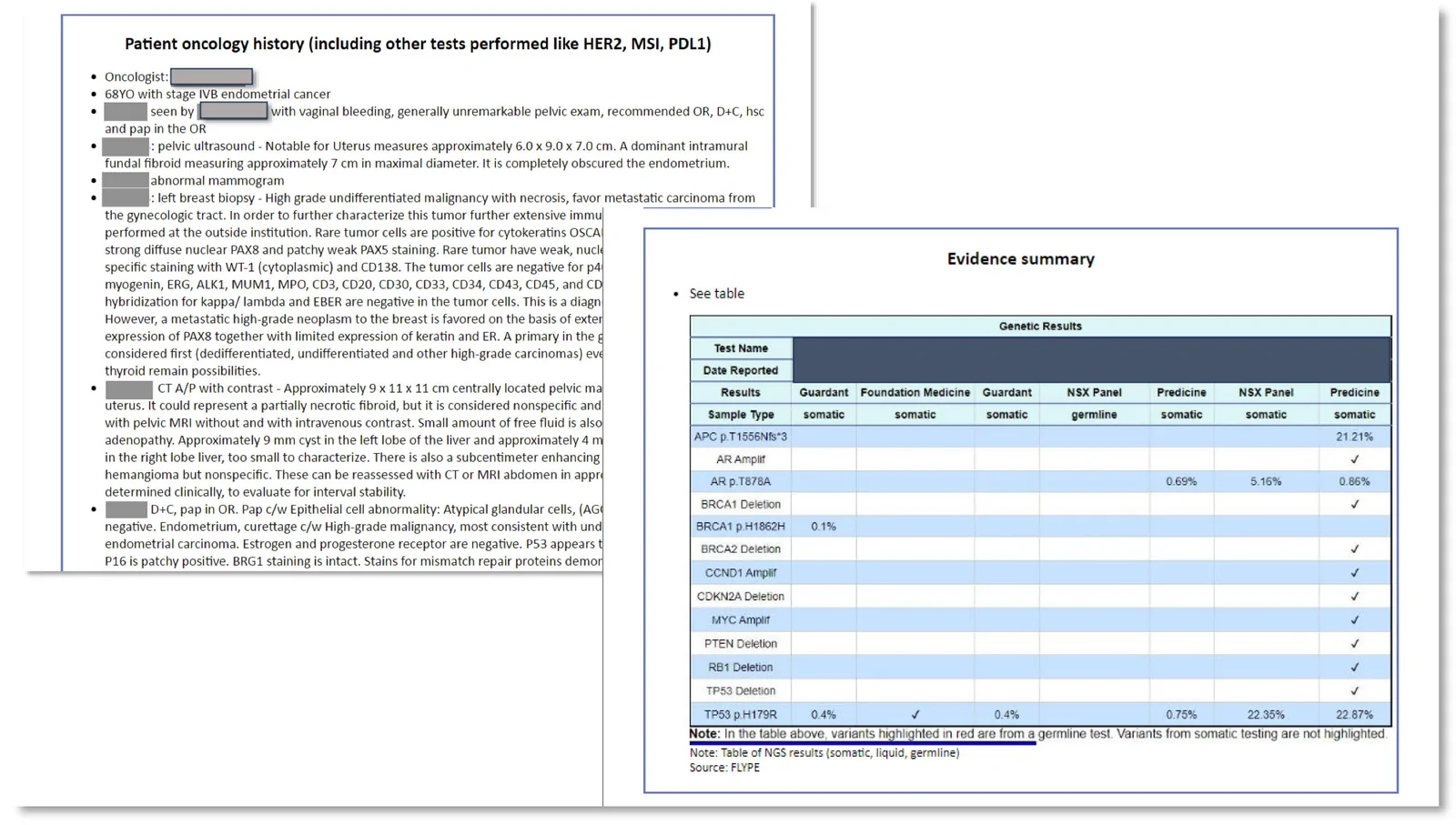
MTB presentation view—The MTB note summarizes patient history, treatment comments and prescribing recommendations (and can include figures). Illustrated in the right panel is an example of a different patient’s history of internal germline and somatic results as well as external testing results obtained from their clinical outcomes page (described below) during their treatment, summarized sequentially.

All NGS samples are reviewed and signed out by credentialed pathologists then sent to our EMR as described [1]. The signed-out results are then available for permissioned advanced practice nurses and pharmacogenomicists to add their interpretive comments and prescribing recommendations for presentation at our molecular tumor board (MTB). Prior to our KCGI [6,9], initial MTB reporting was done through manual chart review and reports generated in Microsoft Word (Microsoft Office LTSC Professional Plus 2021). To streamline report generation and presentation and to simplify integration of results from the reports signed out by Pathology, we added a new module to Flype to build a permissioned MTB report preparation tool that would prepare a summary page to present recommendations to the MTB (Figure 4). A separate knowledge base is available in Flype for MTB note preparation like the Convo tool described in our original Flype manuscript [1] (Figure 5). The MTB report preparation tool also allows image upload to the notes such as illustrative pathway diagrams, figures from manuscripts as well as excerpts of reports (Figure 6).

A total of 431 patients were presented at our MTB from July 2020 to March 2026 [6,9]. Flype’s MTB note preparation tool went live in March 2021, which coincided with the enrollment of the first patients in our KCGI in April 2021. Since the launch of the Flype MTB tool, it has been used to present 319 profiles of patients (a few of which were presented twice or had two NGS specimens for reviewing).

The ability of Flype to integrate multiple internal and external NGS results (below), including our in-house PGX testing of all KCGI patients, allowed better integration of molecular results and better visibility of any potential patient–drug interactions (including pain management) [6,9]. For several patients, the recommendation was made, after reviewing their PGX results, to avoid the use of tamoxifen because they were at an increased risk of treatment failure due to reduced *CYP2D6* activation accompanied by a separate warning that the patient may also receive reduced pain relief from codeine, tramadol, hydrocodone or oxycodone. Other reports included warnings that patients were at an increased risk for thromboembolic events due to variations in the *F5* gene, and some reports contained warnings about an increased risk of adverse effects if treated with thiopurine medications. We found over 40% of our KCGI patients had alterations in typical response to opioids or antidepressants [6]. In addition, over 50% of the KCGI patients were identified as candidates for FDA-approved precision oncology therapies, either on- or off-label. A full discussion of the outcomes of the integration of molecular and PGX testing during our KCGI trial are described elsewhere [6,9].

### 3.3. Clinical Outcomes Integrate Internal and External Molecular Testing with EMR Results

An additional new Flype feature is the development of a Clinical Outcomes module that organizes all molecular testing for each patient, integrating molecular testing from Endeavor Health labs, molecular testing (ctDNA or tissue block) from an ever-growing list of outside vendors (currently Foundation Medicine, Guardant, Predicine, Prevention Genetics and Invitae) along with both Endeavor Health in-house PGX testing and external wellness and PGX panels (Color [1], Sema4, GWA). To support this, we implemented a “third_party” module in Flype to manage external interface queries. Our ability to interface with multiple vendors has been facilitated by requiring vendors to send their code to Redox (Madison, WI, USA) in a standardized format and allowing Flype to retrieve content via the Redox API [13]. Separate Python scripts are run by Linux cron jobs to query Redox periodically and download any new test results from each vendor’s Redox interface. After processing, Flype then forwards the PDF report and any discrete data to our EMR.

The Clinical Outcomes module allows pathologists, clinicians, pharmacists, and APNs to quickly compare a patient’s variants reported from multiple tests over time, including PGX testing and germline testing, without having to toggle between multiple reports in our EMR (Figure 6, right panel). Below the summary table on the patient’s Clinical Outcomes page is a tabular view of each variant or VUS with links to our variant repository’s detail page for those variants, which includes links to other reports with the same variant, as well as links to the original PDF reports for internal and external test results. The Clinical Outcomes module also pulls patient content from our EMR including cancer diagnosis, prescribed oncology drugs and treatments. If the patient has had genetic testing at an external lab, then any FDA-approved on- or off-label targeted therapies recommended by that testing vendor are displayed next to the relevant variant. The Clinical Outcomes module is currently used during MTB report generation, clinical outcomes research and by pathologists to review a summary of the patient’s genetic testing history during their report preparation. We provide a Clinical Outcomes landing page for users whose main role is MTB note preparation.

The Clinical Outcomes report view was also useful during a recent study of metastatic castration-resistant prostate cancer (mCRPC) where treatment was guided by Flype-powered comparisons of internal biopsy NGS tests, germline NGS tests and sequential ctDNA testing in a prospective study at our institution [14]. One example from that study is illustrated in the right panel of Figure 6.

Flype also contains a tool to explore variants observed by gene and diagnosis, then drill down to variant details for any variant previously recorded within its database. This population frequency tool allows users to see how often a particular mutation was recorded within our patient population while also aggregating that information by clinical diagnosis. This allows Flype to report on how many of our patients could be eligible for a clinical trial therapy where the participation criteria include having a certain diagnosis and genetic mutation. Another use case of this tool is to compare our calculated population frequencies against other reported databases (such as COSMIC [15] and gnomAD [16]) to help our internal genetic testing laboratory identify and address quality control issues and prepare for routine inspections.

### 3.4. Managing Variant Annotation

For in-house generated variants, we have updated our annotation pipeline [1] to show MANE Select [17] or MANE Clinical designations from Ensembl’s Variant Effect Predictor (VEP) [18] (VEP111); this helps our pathologists review our chosen transcript designated for each gene and allows our lab to better harmonize our reporting with other institutions. Ensembl also provides UniProt identifiers for most transcripts, which we use to generate the lollipop plot [19] on the variant detail page for exonic variants using InterPro [20] and Pfam [21] domain information showing where the variant occurs within the protein. We added AlphaMissense [22] predictions along with SIFT [23] and PolyPhen2 [24] predictions to our variant detail page and integrate the latest ClinVar [25] record for that position using tabix [26] to help with the interpretation. (We schedule Linux cron jobs to run nightly to download the latest version of ClinVar as soon as it is available, then symbolically link it as “clinvar.vcf.gz” so we always query the latest version for chromosome and position using tabix [26] as the variant detail page is being compiled). We also include a summary of the number of patients where this variant has been observed and include hyperlinks to earlier reports, in case the pathologist wishes to review prior treatments or outcomes. We added a dialog box where pathologists can add a note specific to that variant or flag the variant as noise on the results page and that comment is also displayed on the variant detail page.

We have implemented scripts within Flype’s annotation module to help with the validation process when upgrading to newer versions of Ensembl’s VEP. We keep the legacy VEP annotations active but insert the new VEP annotations into the annotation tables, etc., allowing us to programmatically compare annotations for each variant and identify variants where more than a transcript version upgrade was observed. In a migration from VEP109 to VEP111, we found that 97.96% of our ~300,000 variants required no inspection and our software classified variants requiring manual review. After reviewing our programmatic comparisons and receiving approval, based upon our analysis of discrepant variants, from the Molecular Pathology Director, we ran a structured query language (SQL) script to make the latest annotation live in our database with no additional downtime.

Outside lab results are signed-out clinical results, so we maintain a separate “outside vendor” database table for these results to avoid accidentally reannotating or reinterpreting signed-out clinical results. Some vendors provide complete discrete data with chromosomal coordinates, but others only provide the gene symbol and HGVS [27] pDot or cDot notation (and their preferred transcript in their report’s boiler plate). We capture as much information as the vendor provides and use that to populate our external variant detail page.

### 3.5. Coverage Analysis for New Assay Validation and Sample Reporting

**Figure 7:**
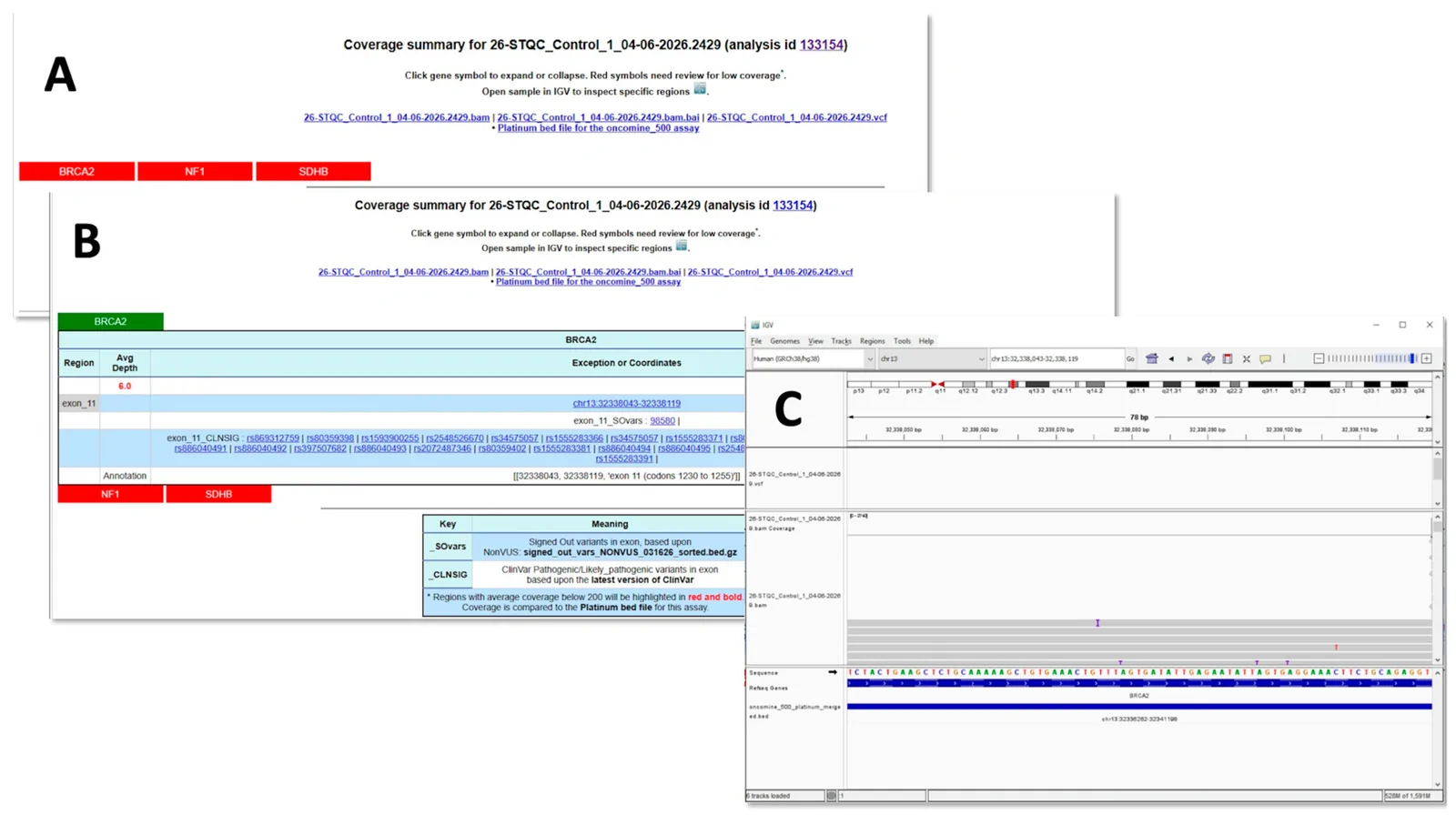
Coverage Summary view showing regions of our Oncomine OCAPlus “platinum” bed file not adequately covered in a control sample. (**A**) The summary view shows genes to review. (**B**) Clicking a gene symbol expands the box to show coordinates, signed out variants and Pathogenic or Likely pathogenic ClinVar variants in that region. (**C**) Clicking the coordinates will take the molecular pathologist to that region in IGV for closer review.

Our laboratory decided to migrate from the Oncomine 161 plus Oncomine 409 assays to the single Oncomine OCAPlus assay for improved throughput. Our team (bioinformaticians, pathologists and the NGS laboratory manager) worked closely with the vendor to launch this assay using hg38 as the reference genome instead of the supported hg19 genome, used for the Oncomine 161 and Oncomine 409 panels, to reduce alignment and annotation errors due to differences between the genome versions. To our knowledge, our institution is the first to validate and use the OCAPlus assay aligned against hg38, requiring independent validation of sample coverage by our team. One set of tools we developed involved building what we termed a “platinum” bed file showing areas of each target oncogene and tumor suppressor gene we successfully sequenced with adequate depth (defined as 200-fold reads at least 80% of the time) across 70 control samples. This allows us to provide clinicians with lists of regions of these target genes we do not routinely cover. In addition, on an individual sample basis, we examine the actual sequence depth of every targeted exonic and intronic region we expect to see in the platinum bed and provide a coverage summary linked to each sample results page showing genes which require closer inspection by the pathologists. For each area with coverage below 200 reads, we identify known “pathogenic” or “likely pathogenic” variants from the current ClinVar [26] as well as variants we have previously signed out from that region. We provide hyperlinks from each set of coordinates which will jump to that region when the patient’s Binary Alignment Map (BAM) file is opened in the Integrative Genomics Viewer (IGV) [28,29] (see Figure 7). We provide copies of the appropriate platinum bed file available for download from the Django static folder along with links to the patient’s BAM and other files for download to the pathologist’s workstation for review in IGV. Regions identified as low coverage in each sample are stored in our SQL database for incorporation into the patient’s signed-out report. We have implemented the same platinum bed analysis and coverage summary analysis for the 84 gene Heme panel, also aligned against hg38.

### 3.6. Convo 2.0—Enhanced Knowledge Base for Reporting

Convo 2.0 is an extension of our earlier Convo knowledge base [1] where we match previously signed-out content for gene/variant/disease with new instances of that gene/variant/disease. We pre-populate the sign-out screen with information from that patient’s sample dashboard, such as “A *KRAS* G12D variant was observed with a frequency of 35% in this patient…” then allow the pathologist to review the current OncoKB entry (licensed for use in reporting and pulled via their API) for that gene/variant/disease combination. We link the diagnosis entered in PowerPath with MSK’s OncoTree [30] for standardization. Integration through Flype using the OncoKB API allows Flype to enable better reporting of OncoKB utilization. Variant interpretations can be manually entered by a pathologist, though new interpretations require review by another pathologist. Convo 2.0 interpretations that have not been reviewed or updated within six months are flagged as “Stale” and need to be updated before use. The need for continuously updating knowledge bases was highlighted in our recent KCGI paper where we noted that, over the course of the KCGI from 2021 to 2024, there were at least 10 new drugs approved that require assessment of a DNA/RNA-based biomarker [6].

Our goal in implementing Convo 2.0 was for the pathologists to be able to sign out more than two or three reports per day per pathologist. With Convo 2.0 and the integration of the OncoKB knowledge base, one pathologist can now handle the workload originally requiring several pathologists to complete in the same amount of time and can sign out nine to ten heme panels or six to seven expanded panels in a day.

### 3.7. Clinical Trials and Submissions to NCI ComboMATCH

Our institution is an Academic Designated Laboratories—Tumor Testing for the National Cancer Institute’s ComboMATCH program [31]. To implement our ability to share patient reports with NCI, we added another interface to our third_party module, used to communicate with outside vendors, allowing Flype to communicate through the NCI’s Data Linkage and Analysis Portal (DLAP) API. This allows us to submit discrete patient molecular data along with diagnostic summaries and retrieve relevant clinical trial recommendations for our patients, which are then integrated into the patient reports generated by Flype. We maintain a local reference file of current criteria for inclusion in active clinical trials (tumor type/gene and sometimes specific amino acid changes) and include information about the clinical trial in reports matching those criteria.

### 3.8. Genetics Counselor Module

In response to requests from our Personalized Medicine colleagues, we added a genetics counselor (GC) module where GCs can enter their patient’s genetic test results manually or link to results downloaded monthly from our vendor. Our motivation with this module is to eliminate the individual spreadsheets of patients and variants each GC maintained, to make that information more widely available within their group and to preserve historical information while giving the GCs access to Flype’s variant repository, automated report generation and other tools. As previously described [1], access is controlled using an individual’s enterprise login followed by project-specific access maintained by the Flype administrators. We provide GCs a custom landing page in their permissioned view of Flype with the ability to navigate to other areas of Flype based upon the access they were granted. The GC module allows management of the patient workflow by grouping patients into the following categories: Initial, New Patient, Appointment Completed, Initial Patient Contact Completed, Testing Ordered (or Testing Deferred), Results Received and Patient Results Disclosed. Clicking a button from the dashboard (such as “Disclosed”) moves patients to the next stage in the workflow. Users can enter patient-specific notes and add non-SNP data such as copy number alterations. The GC module offers menu options to enter a new patient, view “All Cases”, view “My Cases”, reassign a case to another GC as well as the option to browse the external variants database to search for patients with the same variant.

To support referrals of patients with variants that have been flagged for follow-up germline testing per the American Society for Clinical Oncology (ASCO) guidelines, we added a link to the NGS report sign-out page for the pathologist to refer a patient for germline genetic testing and genetic counseling.

We are working with our vendors to fully automate test result retrieval to eliminate the need for any manual variant entry. Until then, GCs are given a simple form to enter their patient’s observed variants. Manually entered GC variants are compared against our existing external variant database before new variants are entered. When detailed molecular information is provided (i.e., GRCh38 coordinates), we can include current ClinVar interpretations as well as gnomAD population frequencies; as described above, we update our local copy of ClinVar weekly to ensure we provide the most recent interpretations, which is especially useful for historical test results.

### 3.9. Other Flype Enhancements

Additional Flype tools such as our algorithms to calculate copy loss and Microsatellite Instability (MSI) from Ion Torrent results will be described in detail in a separate manuscript. The algorithm used to calculate MSI status assesses 24 homopolymer regions across the 409 genes in the Oncomine 409 assay. The data are compared with normal controls to determine a ratio of unstable loci and, if a certain threshold is reached, the tumor is deemed “Microsatellite Unstable”; if within the normal range, it will be reported as “Microsatellite Stable”. This assay approach is currently being validated for inclusion in the Oncomine OCAPlus assay reporting.

## 4. Discussion

Flype is different for every user, based upon their role in NGS reporting, lab assays, PGX, MTB, GC or bioinformatics. All the new Flype features described above have been implemented as separate Django modules as an extension to our previously described Flype software [1]. Unlike tools like SIMPL [10] or Annot [32], which are stand-alone applications, we have integrated our sample tracking system (Docket) with our core Flype software for better interoperability. We recently extended Docket to support FISH sign-out and reporting as a separate module until our institution migrates to EPIC/Beaker.

Since its launch in 2015, Flype has supported the interpretation and sign-out of over 4000 hotspot assays, over 7000 heme and myeloid assays, over 5400 Oncomine 161 assays and over 600 of our recently launched Oncomine OCAPlus assays. Flype has seen over 560,000 variants from internal testing, 12,000 variants from external testing and supports over 170 users with different access privileges. Flype hosts germline test results from our internal 161 panel as well as from external vendors, both in the form of limited 30 gene cancer risk or cardiology risk panels (Color, Sema4), larger panels (Invitae) and cell-free DNA (Predicine). Flype hosts genetic counselor results for almost 13,000 patients.

Since our launch of PGX testing in 2016, Flype has supported the reporting of over 8000 internal pharmacogenomic assays, over 40,000 external pharmacogenomic assays and powers over 450 genomic indicators in EPIC. Our institution has been using genomic indicators in EPIC since 2017 and currently has approximately 175 germline indicators and 140 pharmacogenomic indicators in active use. As discussed elsewhere [12], we have balanced the need to prevent an adverse drug interaction with avoiding “pop-up fatigue” for the providers in the EMR.

### Lessons Learned

Among the lessons learned from Flype’s success within our organization, several key points deserve discussion. First, our bioinformatics team was supported as a stand-alone entity, separate from our institution’s Health IT group but closely aligned with them. As recently described [6], our bioinformatics team has grown over the past ten years from two to five bioinformaticians along with one dedicated Health IT member. Our Health IT group hosts and manages the Linux servers where the Flype software runs, and our team utilizes existing SQL Server databases and domain authentication servers managed by Health IT. Second, our ability to design useful software was user-driven and benefitted from weekly meetings between the bioinformatics team, molecular pathologists, pharmacogenomicists, laboratory managers and medical laboratory scientists. Rather than developing code in isolation, our bioinformatics team would hear a request for a new feature, prioritize it, design a rough implementation and then demonstrate that implementation on our development server at an upcoming weekly meeting for immediate feedback and refinement. Bioinformaticians would openly discuss the assumptions in our software, algorithms and filters and, when the new feature was implemented, would provide documentation the lab could use during regular College of American Pathologists inspections. Third, the bioinformatics team learned to listen to our laboratory and pathology partners as they discussed their pain points. For example, we were able, through implementation of Linux cron jobs, to automate the movement of files from our NGS sequencers to our analysis server and the automated upload of Archer analyses to the separate vendor-provided analysis server. The simple use of scripts and automated processes was able to speed up sample interpretation by at least half a day or more, improving sample turnaround time. Fourth, in addition to openly discussing and documenting features, the enhancement of Flype and its utility to our organization benefitted from the bioinformatics team providing introductory bioinformatics lectures to molecular pathology residents and fellows as well as to our pharmacogenomics residents. We found there is tremendous value to providing some bioinformatics background for the pathologists, pharmacists, genetics counselors and medical laboratory scientists we interact with so they can better follow our discussions, recognize the limitations of our approaches and provide us with better-informed suggestions for new ways to work with the data. Examples of the success of this approach include the impact of our Convo 2.0 with OncoKB on the turnaround time for reporting molecular reports described above (Section 3.6). Finally, another lesson learned is to plan for change. Our institution has received PGX results from five external vendors in addition to our in-house testing. Our in-house testing has utilized two different platforms; we are on the fourth assay update from our current vendor and anticipate migrating to NGS for PGX soon. We had to adapt to changing PGX panels by developing version control for different versions of the reports, in case we needed to regenerate or update a report. We have dealt with changes in our PGX assays by developing separate upload pages for different versions, submitting new assay results flagged as “Validation” while continuing to upload patient results from the current assay. We modified our Kensa [1] software and PGX translation tables to incorporate activity scores in our reporting when recommended by CPIC [33]. We had to develop mechanisms to allow updates from our external vendors, for example to a Hereditary Cancer panel or Cardiac Risk panel, to be transmitted as an update to the EMR with new discrete data being captured; updated outside vendor reports trigger email notifications to the bioinformatics team as well as pharmacogenomics or medical genetics as appropriate. To help manage change, we include both the Flype version and VEP version in reports signed out through Flype. To ensure updates to annotation did not affect previously signed out samples and to help establish audit trail capabilities, we implemented a locked dashboard and write signed-out variants to a JavaScript Object Notation (JSON) file for each sample. A similar theme of the need for planning for change in pharmacogenomic data was shared with us recently by Dr. Robert Freimuth based upon their experiences implementing PGX at the Mayo Clinic (Personal communication).

End user feedback:

The development of Flype benefits from continuous feedback from our end users: the molecular pathologists, pharmacogenomicists, laboratory managers, medical laboratory scientists and oncology advanced practice nurses. Their feedback on current features, suggestions for new features and identification of current needs has been invaluable. In addition, the bioinformatics team relies on guidance from a Bioinformatics Steering Committee that meets two or three times a year, with a department chair and section heads to help validate and guide our team as we prioritize major new initiatives.

Examples of the success of this approach include the identification by molecular pathology that signing out reports was taking too long. This led to the development of Convo 2.0 (Section 3.6, above), with the molecular pathologists advocating for the expense needed to license OncoKB because they saw the value. Discussions with the lab manager and molecular pathologists about uncertainty in the coverage of the new Oncomine OCAPlus panel and challenges in manually reviewing each sample led to the development of coverage algorithms and the ability to manually inspect areas flagged in individual samples using IGV (Section 3.5, above). The development of Docket was in direct response to concerns about sample tracking and managing workflows from the laboratory manager and medical laboratory scientists. The bioinformatics team worked with them weekly to ensure Docket had the necessary features, exported accurate CSV files for use during their assays or to upload to NGS sequencers or other applications. As mentioned above, the successful launch of Docket led the medical laboratory scientists to suggest adding a new Docket-like feature to help support FISH sign-out and reporting as a separate module until our institution migrates to EPIC/Beaker.

Limitations and continued pain points:

As Flype continues to evolve in our institution, we recognize there are still limitations to our implementation. First, many of our new features, such as the ability to provide a Spanish-language translation of a patient’s PGX report, are dependent upon new features yet to be implemented in our institution’s EMR. Our Health IT organization is nearing completion of a multi-year harmonization of EPIC installations across our expanded organization. The complexity of this migration has led to delays in certain features, such as adding a patient’s preferred language to their profile, which have delayed the launch of some new features in Flype. Similarly, implementation of EPIC’s Beaker, when launched, will require some revisions to our Docket architecture. Additionally, we are currently reliant on external testing labs sending results to Redox, but eventually we will need to update our protocols once vendors can provide secure HL7 communication, for example. We also must monitor and manage the continued improvement in software offered by our vendors. For example, we currently download ArcherDx results from the vendor-provided web portal (a locally hosted virtual machine) then upload those results to Flype, but a newer version of the vendor’s software provides an API where we could programmatically pull results into Flype. This has the potential to improve the efficiency of incorporating ArcherDx results but will require development time and validation. Also, we are dependent on the performance of annotation software (Ensembl’s VEP), which can take 30 to 45 min to annotate an Oncomine OCAPlus run and insert the results into our variant database, even though we only annotate new variants we have not seen before. Finally, much of our bioinformatics software is customized for working with Ion Torrent variant call files and will need modification and validation as we begin analyzing patient samples on other platforms.

## 5. Conclusions

In conclusion, Flype has evolved since our last report [1] and was critical in the success of our recently completed KCGI study [6,9]. New features, such as our Clinical Outcomes patient summary, were key to monitoring patient’s tumor progression during a longitudinal study of mCRPC [14]. This study demonstrates how a small team of bioinformaticians, working closely with their molecular pathology colleagues and supported by a strong institutional Health IT organization, can develop software tools to improve the efficiency and turnaround time in reporting by pathology and impact patient outcomes. Developing foundational tools such as Docket, described here to address a significant pain point in our molecular laboratory, has supported higher molecular testing throughput. Flype’s ability to integrate internal and external molecular test results, as well as internal and external pharmacogenomic results, has helped focus patient treatments based upon their ability to metabolize medications instead of relying on trial and error.

## Data Availability

As recently described [6], the enhancements and customizations to the original Flype architecture described in this document, including the development of Docket as a laboratory information system, limits the ability of individual modules of Flype to be used in other settings. If interested, please contact the corresponding author to inquire about licensing a copy of Flype for your institution. The availability of KCGI patient data is discussed in separate manuscripts [6,9].

## Abbreviations

The following abbreviations are used in this manuscript:

API	Application programming interface
APN	Advanced practice nurse
BAM	Binary alignment map
CSV	Comma-separated values
ctDNA	Circulating tumor DNA
EMR	Electronic medical record
FISH	Fluorescence in Situ Hybridization
GC	Genetic counselor
JSON	JavaScript Object Notation
KCGI	Kellogg Cancer Genomic Initiative
LIMS	Laboratory information management system
MRN	Medical record number
MSI	Microsatellite instability
MTB	Molecular tumor board
NGS	Next-generation sequencing
PGX	Pharmacogenomics
TSV	Tab-separated values
SNP	Single nucleotide polymorphism
SQL	Structured query language
VEP	Variant Effect Predictor
